# Subtotal gland resection for MR negative Cushing disease with no detectable tumor on gland exploration: operative video

**DOI:** 10.3171/2023.4.FOCVID2320

**Published:** 2023-07-01

**Authors:** Jamie J. Van Gompel, Irina Bancos, Garret Choby

**Affiliations:** Departments of 1 Neurosurgery,; 2 Endocrinology, and; 3 Otolaryngology–Head and Neck Surgery, Mayo Clinic, Rochester, Minnesota

**Keywords:** Cushing disease, pituitary adenoma, neurosurgery

## Abstract

MRI–Negative Cushing disease is a very difficult disease to treat medically and surgically. In the past, after negative gland exploration, hemihypophysectomy was commonly performed on the localizing side of inferior petrosal sampling. However, this generally resulted in 50% remission/cure rates. Therefore, other techniques have arisen based on the percent chance of microadenoma tumor being present in the gland. Subtotal gland resection is a technique aimed at removing 75% of the gland that results in a similar chance of remission and a 10% chance of pituitary dysfunction. In this video, the authors demonstrate this important technique for MRI–Negative Cushing disease.

The video can be found here: https://thejns.org/doi/abs/10.3171/2023.4.FOCVID2320

## Transcript

Here we present a case of subtotal gland resection for MR-negative Cushing disease with no detectable tumor on gland exploration: an operative video.

### 0:33

This is a 40-year-old woman with ACTH-dependent Cushing syndrome complicated by diabetes mellitus, obesity, and hypertension. Her BMI was 47.5. MRI demonstrated no discrete lesion within her pituitary gland. IPSS was inconclusive and repeated twice.^1,2^ Dotatate PET did not demonstrate any peripheral lesions.^3^ CT chest and abdomen had no lesions in addition.

### 0:57

Here is imaging: outside 3T dynamic MRI was negative, demonstrating a presellar type sella with no evident tumor to be seen or found in this particular case.

### 1:07

We repeated a 7T MRI here with no additional lesions identified.

### 1:13

Operation was performed in a supine position. We used CTA for stereotaxis; binostril technique was utilized. We had a planned subtotal gland resection if there was no tumor found in exploration. TXA^4^ was previously described and our protocol was used.

### 1:28

My friend Dr. Lillehei described this technique in 1989 to 2011 where 22 patients were treated with subtotal gland resection. Eighty-two percent of patients went into remission.^5^

### 1:42

As an alternative, we moved away from IPSS-guided hemihypophysectomy, which in our hands resulted in remission around approximately 50% remission. Recall IPSS can drain contralaterally in some cases, resulting in mislateralization.

### 1:56

Here we are operating through the naris, and posterior septectomy is performed and a wide expanse of sphenoidotomy is performed.

We drill out the gland as well as we can, although we don’t spend excessive amounts of time drilling out the whole inferior clivus. It is important, however, still to be with bone work cavernous sinus to cavernous sinus as we are here. Utilizing a caudal elevator, it is helpful to feel from the paraclival carotid to paraclival carotid to ensure there is no additional bone work that is necessary.

Once this is performed, we carefully open the dura, ensuring not to violate the pseudocapsule or capsule around the pituitary gland in order to explore completely. This is critical especially in Cushing patients. This is a case from approximately 4 years ago. So, no intraoperative ultrasound was utilized to attempt to identify the tumor. Further, this is before my adaptation of medial wall of cavernous sinus resection.

### 3:09

Here we are taking our time to still look for a tumor and as typically is the case even despite the TXA we see bleeding from the circular sinus. We take our time to push the gland away from the interior dura and inspect that surface through a curette.

### 3:30

It is helpful also to take a right-angle hook to feel the gland for abnormalities and expand your dural openings.

### 3:37

Here is the technique of subtotal gland resection as described by Dr. Lillehei, in which you divide the anterior glands into thirds and cut back from the anterior aspect as well as the inferior 125%, preserving a block of pituitary gland in the middle portion of the gland as well as a posterior gland in order to avoid DI and postoperative complications. This technique has approximately a 10% chance of pituitary failure.

### 4:04

Here we see side to side and make our cuts in one-third and an inferior cut.

### 4:11

Here we have some milky white tissue coming out commonly with these cuts, which was a problem with prior exploration. You see this milky white substance come out, which can fool you to be pituitary tumor.

### 4:25

We then take this dissector and work to the back of the pituitary gland, feeling those posterior clinoids. I’m also suspicious that we see a tumor midgland but continue with the plan of subtotal gland resection. I do not explore dramatically in these cases. I take a pituitary right-angle curette and take these pieces out and allow the pathologists examine postoperatively. We try to take them out in larger pieces. So that’s a portion of that lateral gland. There is a little, but more posteriorly, so we’ll continue to work that plane in order to provide less manipulation and damage to the intact block we plan to keep behind.

### 5:09

Here again, we are going to take another separate piece out and keep that as the right side of the gland. We are also going to inspect inferiorly and then to the left, and we’ll take this out as a separate specimen on the left side.

### 5:23

In a similar technique, and you’ll see again that milky white tissue come out that people believe is tumor sometimes. That’s simply necrotic or infarcted gland from your compression of the gland. Now we are going to send this now as left gland.

### 5:42

You’ll see here in the middle portion as we explore that left cavernous sinus wall there appears to be a tumor evident, just above the sucker currently. We are going to continue and look and see if there is anything attached to the cavernous sinus walls. Here we’ve also removed that inferior block and set that as a separate specimen. We are going to continue to explore the gland; however, right here we are going to start to become suspicious of a tumor right in the middle portion of the gland. Kind of see a whitish tissue extending just above our sucker.

### 6:20

You can see now there is some very clear different type of material coming out, and we’ll find a pseudocapsule right here, and you can see a tumor right there in the middle in our field. Ultimately in pathology, this will prove to be the tumor.

### 6:39

We are going to stop and rather than do a pseudocapsular technique, I want to ensure, because I’ve already violated, that we have pathologic tissues, so we’ll send this as a separate specimen despite already having left, right, and inferior gland specimens. You see how white and different that is from the rest of that yellowish gland. Now again, in Cushing disease the gland is oftentimes very yellowish from the Crooke’s changes that we see.

### 7:08

Now we are going to inspect that cavity and try to take out the pseudocapsule width. Some additional gland and try to get a margin plus resection in this particular circumstance. There is an additional portion of inferior gland posteriorly that we will remove as part of our plan of subtotal gland resection right here.

### 7:40

Again, we try to remove these all in separate blocks. At this time as well, we are going to continue to—after we believe we have complete exoneration of that capsule—we are going to take a lot of time to again inspect for separate tumors. It is not uncommon to see more than one tumor in these cases. We have no CSF leak, as can be seen, although you can see the diaphragm off to the left there. We are going to look through that right-angle curette. You can see how the subtotal gland resection allows you to really provide a lot of room within the pituitary sella itself to allow inspection and that bleeding is now controlled.

### 8:19

It is important in this first operation to be very thorough and take your time, as we are here.

I try to do minimal manipulation with the posterior gland. You can see that yellowish posterior gland on the inferior left-hand side—we were just down by it. Again, it is going to be on the back side of the left side. It isn’t uncommon for us to submarine here.

### 8:47

Here I’m trying to ensure—you can see that area of gland that resected adjacent to that prior tumor—I’m trying to ensure there is no additional tumor in that location. There is the posterior gland again, right there, right to the back side right there; you can see the medial wall of cavernous sinus dura really well. Then we’re just going to take some liquid Gelfoam and fill the cavity and put some bloody Gelfoam in there. Nowadays, we would place a floor flap in there as well, but this is adequate closure for this.

### 9:23

This demonstrates all the other specimens being negative; however, there was tumor found as we showed.

Postop day 1, the cortisol was 1.5 µg/dl, proving cure.

Dismissed postop day 2.

Three years out after surgery, she remains in remission and cure. She had postop adrenal suppression for between 6 and 12 months, diabetes improved, and hypertension is resolved.

### 9:45

Here is what an MRI looks like after this. You can see a fair amount of pituitary tissue done, but the pituitary gland is intact.

I have found this subtotal technique very helpful in my practice. There appears to be low complication rates with this. However, I will emphasize that I do not perform this on patients contemplating pregnancy in the future.

Here are some additional references.

We appreciate your time.

## Disclosures

Dr. Bancos reported consulting fees from Recordati, Corcept, HRA Pharma, Sparrow, Adrenas, Neurocrine, Spruce, and Diurnal, outside the submitted work.

## Author Contributions

Primary surgeon: Van Gompel, Choby. Editing and drafting the video and abstract: Van Gompel, Bancos. Critically revising the work: all authors. Reviewed submitted version of the work: all authors. Approved the final version of the work on behalf of all authors: Van Gompel. Supervision: Choby.
